# Transformation induced by Ewing's sarcoma associated EWS/FLI-1 is suppressed by KRAB/FLI-1

**DOI:** 10.1038/sj.bjc.6600669

**Published:** 2003-01-28

**Authors:** D Chan, T J Wilson, D Xu, H E Cowdery, E Sanij, P J Hertzog, I Kola

**Affiliations:** 1 Centre for Functional Genomics and Human Diseases, Monash Institute of Reproduction and Development, Monash University, Melbourne, Australia; 2 7245-24-110, Pharmacia and Upjohn, 301 Henrietta Street, Kalamazoo, MI 49007, USA

**Keywords:** Ewing's sarcoma, repressor, EWS, KRAB, Fli-1

## Abstract

Ewing's sarcoma is a childhood bone tumour with poor prognosis, most commonly associated with a t(11;22)(q24;q12) reciprocal translocation that fuses the *EWS* and *FLI-1* genes, resulting in the production of an aberrant chimeric transcription factor EWS/FLI-1. To elucidate the mechanisms by which EWS/FLI-1 mediates transformation in mouse models, we have generated a murine Ews/Fli-1 fusion protein. We demonstrate that this protein transforms fibroblast cells *in vitro* similar to human EWS/FLI-1 as demonstrated by serum and anchorage-independent growth, the formation of tumours in nude mice and elevation of the oncogenic marker c-myc. Furthermore, transformation of these cells was inhibited by a specific repressor, KRAB/FLI-1. The KRAB/FLI-1 repressor also suppressed the tumorigenic phenotype of a human Ewing's sarcoma cell line. These findings suggest that the transformed phenotype of Ewing's sarcoma cells can be reversed by using the sequence-specific FLI-1-DNA-binding domain to target a gene repressor domain. The inhibition of EWS/FLI-1 is the first demonstration of the KRAB domain suppressing the action of an ETS factor. This approach provides potential avenues for the elucidation of the biological mechanisms of EWS/FLI-1 oncogenesis and the development of novel therapeutic strategies.

Ewing's sarcoma (ES) and primitive neuroectodermal tumours (PNET) belong to the Ewing family of tumours, which is a subgroup of small-round-cell tumours (Ewing, 1984). The clinical prognosis of individuals with these tumours is poor because of the lack of specific therapies and a high incidence of relapse (Kovar *et al*, 1990; Ambros *et al*, 1991). Monitoring the disease is also difficult because of the absence of specific phenotypic markers. The majority of ES/PNET have been shown to have a t(11;22)(q24;q12) chromosomal translocation that fuses the amino terminus of the *EWS* gene to the carboxyl terminus of the *FLI-1* gene (Delattre *et al*, 1992).

The fusion of an Ets DNA-binding domain (DBD) to EWS appears to be an important determinant in the generation of ES/PNET. While 85% of ES/PNET translocations involve *FLI-1*, another 5% involve *ERG*, a closely related ETS transcription factor and other rarer translocations in ES have included only closely related members of the ETS family. While human EWS/FLI-1 fusion is capable of transforming, neither the N-terminal *EWS* nor FLI-1 DBD domain transformed NIH3T3 cells (May *et al*, 1993a; Lessnick *et al*, 1995). This suggests that the EWS/FLI-1 fusion protein does not act by blocking the binding of normal cellular FLI-1 and is consistent with the role of EWS/FLI-1 as a novel oncogenic transcription factor. Consistent with this hypothesis, in ES/PNET tumour cell lines or human EWS/FLI-1-transformed NIH3T3 cells many genes associated with tumorigenesis have also been shown to be upregulated including c*-myc, stromelysin-1, Manic Fringe, mE2C* and *EAT-2* (Bailly *et al*, 1994; Braun *et al*, 1995; Thompson *et al*, 1996; May *et al*, 1997; Arvand *et al*, 1998; Dauphinot *et al*, 2001). Since EWS/FLI-1 or EWS/GAL4 can more efficiently activate reporter constructs regulated by HTLV Ets DNA-binding or GAL4 DNA binding elements than similar FLI-1 proteins (May *et al*, 1993b; Bailly *et al*, 1994), it has been suggested that EWS/FLI-1 could act by overexpressing genes normally regulated by FLI-1, resulting in tumorigenesis.

Although the *EWS/FLI-1* fusion gene is capable of acting as an oncogene, the mechanism that results in the EWS/FLI-1 translocation in ES/PNET and the direct effects of the resultant protein are unknown. Indeed, the EWS/FLI-1 fusion protein may also have alternate activities since mutation of the Ets DBD does not ablate all transforming abilities and EWS/FLI-1 can also affect mRNA splicing (Jaishankar *et al*, 1999; Knoop and Baker, 2001; Welford *et al*, 2001). While the use of antisense oligonucleotides or transcripts to inhibit EWS/FLI-1 expression or suppression of a variety of signalling pathways reduced the tumorigenic potential of ES/PNET cells (Ouchida *et al*, 1995; Kovar *et al*, 1996; Tanaka *et al*, 1997; Toretsky *et al*, 1997), these reports confirm the central role of EWS/FLI-1 in ES/PNET, but do not address the mechanism by which EWS/FLI-1 acts. Since cancer rarely results from a single mutation and, like other cancers, ES/PNET cells contain other mutations (e.g. p53), understanding the precise mechanism by which the EWS/FLI-1 protein results in transformation will be important in the development of effective therapies for this condition.

One approach to inhibit only the transcriptional activity of EWS/FLI-1 is to introduce specific transcriptional suppressors into EWS/FLI-1-transformed cells that can bind and actively suppress EWS/FLI-1 target genes. The Kruppel associated box (KRAB) is a protein domain of approximately 75 amino-acid residues and has been shown to act as a potent DNA-binding-dependent transcriptional repressor (Margolin *et al*, 1994; Witzgall *et al*, 1994; Pengue *et al*, 1995; Vissing *et al*, 1995) that inhibits the transcriptional machinery and/or alters the chromatin structure (Kingston *et al*, 1996; Moosmann *et al*, 1997). Approximately, one-third of the zinc-finger proteins of the Kruppel Cys_2_His_2_-type contain KRAB domains at their amino termini (Bellefroid *et al*, 1991). Recently, constructs linking KRAB to a variety of DBD have been shown to efficiently suppress the activity of promoter–reporter constructs in a DNA-binding specific manner (Beerli *et al*, 1998; Herchenroder *et al*, 1999; Ma *et al*, 1999; de Haan *et al*, 2000). Furthermore, the introduction of KRAB fused to PAX3 or Myb DBD into rhabdomyosarcoma cells or haemopoietic tumour cell lines, respectively, can inhibit their transformed phenotype (Rossi *et al*, 1999; Ayyanathan *et al*, 2000; Fredericks *et al*, 2000; Nawrath *et al*, 2000).

Thus, we have generated a construct that contains KRAB linked to the DBD of FLI-1 and examined its ability to reverse the phenotype of human ES/PNET cell lines or NIH3T3 cells transformed by both human or mouse EWS/FLI-1 oncogenes. We have demonstrated that KRAB/FLI-1 expression reduces the ability of these cells to proliferate in low serum, form colonies in soft agar and tumours in nude mice and reduced the upregulation of *c-myc*. The initial transformation by EWS/FLI-1 and reversal by KRAB/FLI-1 were similar for both mouse and human genes and demonstrate that the generation of a mouse model for this disease by manipulating the mouse genome is appropriate for elucidating the mechanisms and validating novel therapeutic strategies. Furthermore, these data demonstrate that inhibition of EWS/FLI-1 transcription via sequence-specific DNA binding of the KRAB repressor domain is sufficient to inhibit the transformed phenotype. Therefore, targeted gene repression may be a potential approach for the elucidation of the biological mechanisms of EWS/FLI-1 oncogenesis and the development of novel therapeutic strategies.

## MATERIALS AND METHODS

### Plasmids

**Figure 2:**
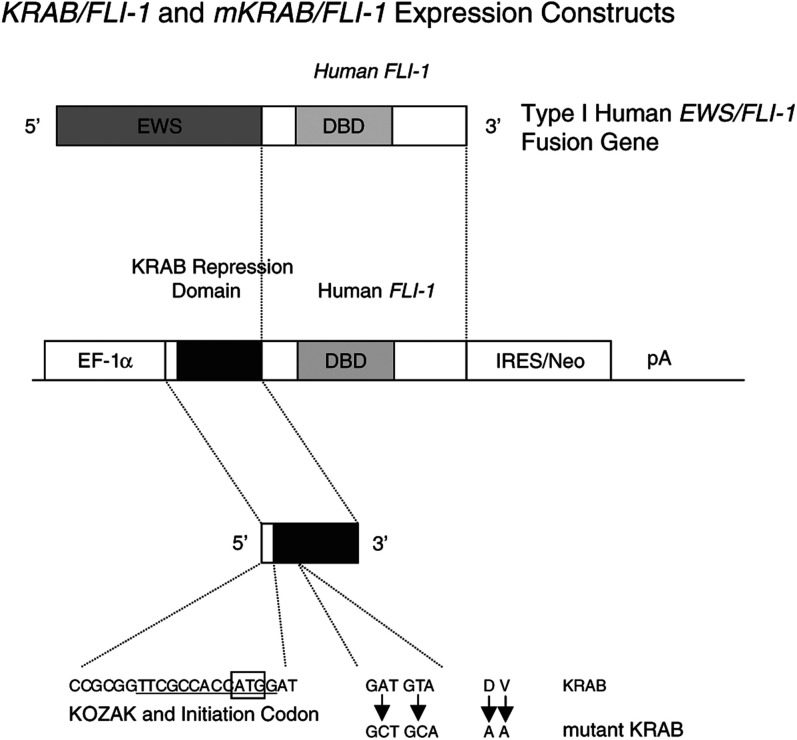
Schematic diagram of KRAB/FLI-1 and mutant KRAB/FLI-1 expression constructs. Either KRAB or mutant KRAB with an engineered KOZAK consensus sequence with initiation codon, ATG, was used to replace the *EWS* transactivation domain in the human type I *EWS/FLI-1* fusion gene. The elongation factor-1α promoter was used to express *KRAB/FLI-1* or mutant *KRAB/FLI-1* and an IRES/Neo-resistance gene.

The murine *Ews/Fli-1* fusion gene was designed to recapitulate the human type 1 *EWS/FLI-1* fusion sequence. The murine *Ews/Fli-1* fusion cDNA construct was generated by first amplifying exons 1–7 on murine *Ews* cDNA and exons 6–9 of murine *Fli-1* cDNA, and then fusing both partial cDNAs together by using an overlap extension PCR technique. The murine *Ews/Fli-1* and human type 1 *EWS/FLI-1* fusion genes were cloned into the pEF-BOS vector (Mizushima and Nagata, 1990) containing a *puromycin* resistance gene. Similar constructs were also generated using the cytomegalovirus (CMV) minimal promoter. The *KRAB/FLI-1* fusion gene was made by replacing *EWS* cDNA (amino acids 1–244) in human *EWS/FLI-1* type I fusion cDNA with a KRAB domain derived from KOX1 (Figure 2

). Mutant KRAB (amino acids 18 and 19 were changed from DV to AA) was used as negative control because the two substitutions abolish binding to the corepressor KAP-1 and thus the KRAB repressor function (Friedman *et al*, 1996; Moosmann *et al*, 1997; Figure 2). A KOZAK consensus sequence and initiation codon was added to KRAB and mutant KRAB by PCR amplification using the oligonucleotide; 5′-TCCCCGCGGTTCGCCACC**ATG**GATGCT-3′. A promoterless IRES/Neo/PolyA was cloned behind the *KRAB/FLI-1* or *mutant KRAB/FLI-1* fusion genes to allow selection of clones with high expression.

### Cell culture and transfection

NIH3T3 cells were maintained in Dulbecco's modified Eagle's medium (DMEM; GIBCO) supplemented with 10% fetal calf serum (FCS; Life Technology), 3.7 g l^−1^ of sodium bicarbonate (BDH) and 10 mM penicillin/streptomycin. SK-N-MC cell line was obtained from the American Type Tissue Collection (ATCC) and was cultured in Eagle's minimal essential medium (EMEM; GIBCO) supplemented with 10% FCS, 0.1 mM nonessential amino acids (GIBCO), 1.0 mM sodium pyruvate (GIBCO), 1.5 g l^−1^ sodium bicarbonate (BDH) and 10 mM penicillin/streptomycin. All cell lines were grown in 5% CO_2_. To examine growth of cell lines in low serum, the cell lines were washed twice in phosphate buffered saline (PBS) and placed in media supplemented with 1% FCS.

To establish cell lines that stably express murine or human EWS/FLI-1, 10 *μ*g of each EWS/FLI-1 expression constructs was electroporated into 1×10^6^ NIH3T3 cells in 0.1 ml culture medium at 650 mF and 200 V in a 0.4 ml cuvette with the BIO-RAD Gene Pulser and Pulse Controller Transfection apparatus. Promoter constructs without the fusion protein were similarly transfected as ‘mock’ or normal controls. The transfected cells were selected with 5 *μ*g ml^−1^ puromycin for 1 week. The puromycin-resistant colonies were cloned, expanded, cryopreserved and evaluated for expression of protein by Western analysis. Clones of EWS/FLI-1-transformed cells were similarly transfected with *KRAB/FLI-1* or *mutant KRAB/FLI-1*, selected with 400 *μ*g ml^−1^ G418 for 2 weeks and treated as described above.

### Antibodies and Western blot

Total cell lysates were prepared from cells at 70% confluence and electrophoresed on an 8% denaturing sodium dodecyl sulphate–polyacrylamide gel electrophoresis (SDS–PAGE) gel. The proteins were electroblotted onto Hybond C extra membranes (Amersham). The filters were blocked in 10% skim milk powder (Sunshine) dissolved in TBST (20 mM Tris, 137 mM NaCl, 0.1% Tween 20, pH 7.6) for 1h at room temperature. Incubation with primary antibodies were carried out at 4°C overnight. Antibodies used were anti-FLI-1 (Santa Cruz; detects EWS/FLI-1, KRAB/FLI-1 and mutant KRAB/FLI-1), c-myc (Santa Cruz) and *α*/*β* tubulin (Boeringer Mannheim). The secondary antibody conjugated to horseradish peroxidase was detected using the ECL Western Blotting Analysis System (Amersham) and exposed to Kodak X-AR film.

### Total RNA extraction

Total RNA from each of the cell lines was prepared by harvesting the cells when they were at 70% confluency. Approximately, 1×10^6^ cells were washed twice with PBS and total RNA extracted using the High Pure RNA Isolation kit (Roche) as described by the manufacturer and then quantified by absorbance at OD_260_ using a PE spectrophotometer (Perkin-Elmer).

### Cell proliferation assay

The growth rate of each cell line was examined in media containing 1% FCS. NIH3T3 (5×10^3^ cells) or SK-N-MC (1×10^3^ cells) were seeded in triplicate in 3 cm wells in low serum media and cell proliferation measured by direct counting of trypan-blue negative cells.

### Soft agar assays

In all, 5000 cells of each cell line were trypsinized and suspended in 2 ml of complete medium plus 0.3% agarose (Promega). The agar–cell mixtures were plated on top of a bottom layer with 1% complete medium agar mixture in triplicate. After 2–3 weeks, the agar assays were scored for viable colonies.

### Mouse tumour formation assay

The tumorigenic potential of each cell line was evaluated by injecting of cell suspensions into 2- to 3-week-old *BALB/c nu/nu* female mice. For each clone tested, five mice received subcutaneous injections of 2×10^6^ cells (NIH3T3 cell lines) or 5×10^6^ cells (SK-N-MC cell lines) in a volume of 0.2 ml of PBS into both dorsal midline sides. The mice were housed in microisolator cages, given food and water *ad libitum*, and handled in a sterile laminar-flow hood. Tumour sizes were measured every 3 days using Vernier calipers along two perpendicular axes. The volume of the tumours was calculated by using the following formula: (mean diameter)^3^×*π*/6. All animal experimentation was performed with approval from the Monash University Ethics Committee and conformed to UKCCCR guidelines for the Welfare of Animals in Experimental Neoplasia (UKCCR, 1998).

## RESULTS

### Transformation of NIH3T3 cells by murine or human EWS/FLI-1 fusion genes

**Figure 1:**
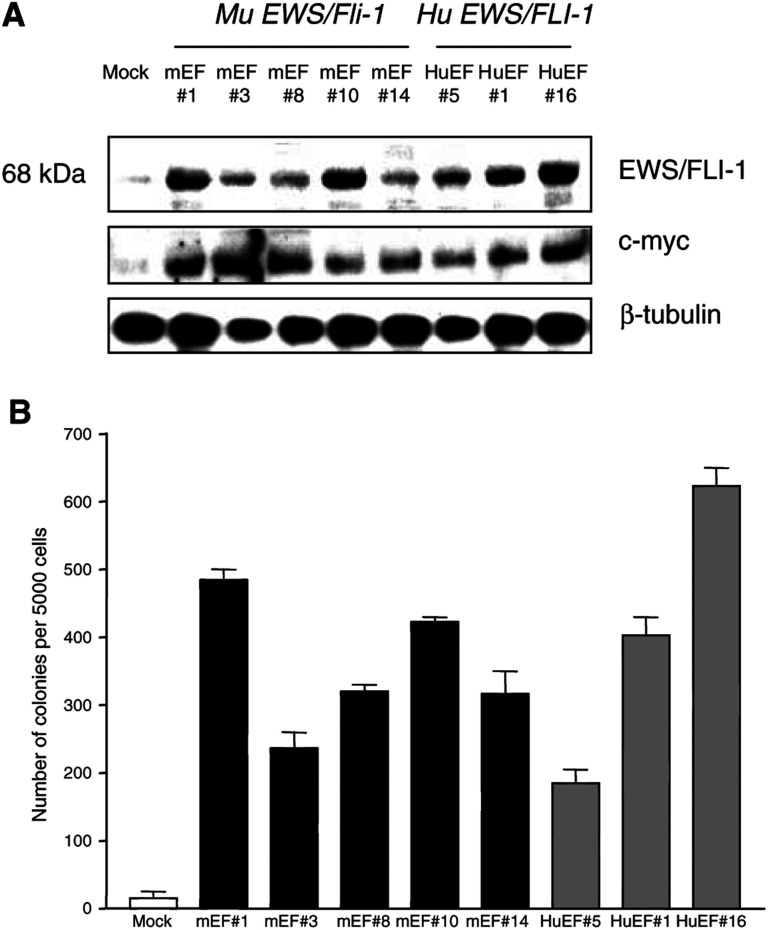
Expression and tumorigenicity of murine and human *EWS/FLI-1*. (**A**) Western blot showing EWS/FLI-1 and c-myc protein expression in murine and human EWS/FLI-1-transformed cells. EWS/FLI-1 (68 kDa) was detected by Fli-1 (C-19) polyclonal antibody and c-myc protein by c-myc (N-262) polyclonal antibody. β-tubulin protein was used as a loading control. (**B**) Soft-agar assay on murine and human EWS/FLI-1-transformed NIH3T3 cell lines. In all, 5000 cells of each cell line were grown for 12 days in a medium containing 0.3% agarose and colonies >20 cells counted. Values shown are the mean ± s.e.m. of triplicate samples.

To determine whether murine Ews/Fli-1 is capable of transforming cells in a similar manner to that of the human *EWS/FLI-1* fusion gene, we generated stable NIH3T3 cell lines transfected with mouse or human *EWS/FLI-1*. Clones were selected with puromycin and testing for mRNA expression by RT–PCR (data not shown). Positive clones were demonstrated to express the expected 68 kDa EWS/FLI-1 band on Western blot analysis using a polyclonal antibody to the C-terminal of the Fli-1 protein (Figure 1A

). Cells transfected with the empty vector were used as controls, which displayed a weak nonspecific band that has been observed with this commercial antibody in other studies (May *et al*, 1997). Both human and mouse EWS/FLI-1 transfectant clones displayed a variety of levels of EWS/FLI-1 fusion protein expression.

One of the hallmarks of the transformed phenotype is the ability of cells to grow in semisolid medium (Rizzino, 1987). At least three cell lines from each genotype were tested in triplicate with data from representative clones shown (Figure 1B). The control cell lines grew poorly in soft agar with only 15±10 small colonies (<20 cells) per 5000 cells plated observed. In contrast, all human EWS/FLI-1 and murine Ews/Fli-1-expressing clones grew efficiently in soft agar, forming large macroscopic colonies of greater than 200 cells, indicating anchorage-independent growth (Figure 1B). For example, human EWS/FLI-1-transformed clone HuEF#1 formed 403±26 colonies/5000 cells and mouse Ews/Fli-1 clone mEF#14 formed 317±33 colonies/5000 cells. The efficiency of colony formation was similar for cell lines expressing mouse or human EWS/FLI-1. Moreover, the level of expression of the fusion protein appeared to correspond to the efficiency of colony formation (Figure 1A and 1B). For example, HuEF#16 formed 623±27 colonies/5000 cells, whereas HuEF#1, which expressed less EWS/FLI-1, only formed 403±27 colonies/5000 cells. These data demonstrate that murine EWS/Fli-1 is capable of transformation similar to human EWS/FLI-1 and may indicate that the amount of EWS/FLI-1 oncoprotein is proportional to the proliferation rate and potency of transformation of NIH3T3 cells.

To determine whether *c-myc*, which is one of the genes known to be activated in ES/PNET tumour cells, was also altered in our human and murine EWS/FLI-1-transformed NIH3T3 cells, we examined the expression levels of c-myc by Western blot (Figure 1A). Levels of c-myc were elevated in both mouse and human *EWS/FLI-1*-transfected cells compared with controls, demonstrating that human and mouse EWS/FLI-1 activate a similar downstream signalling cascade.

### Expression of the KRAB/FLI-1 repressor in EWS/FLI-1-transformed NIH3T3 cells

**Figure 3:**
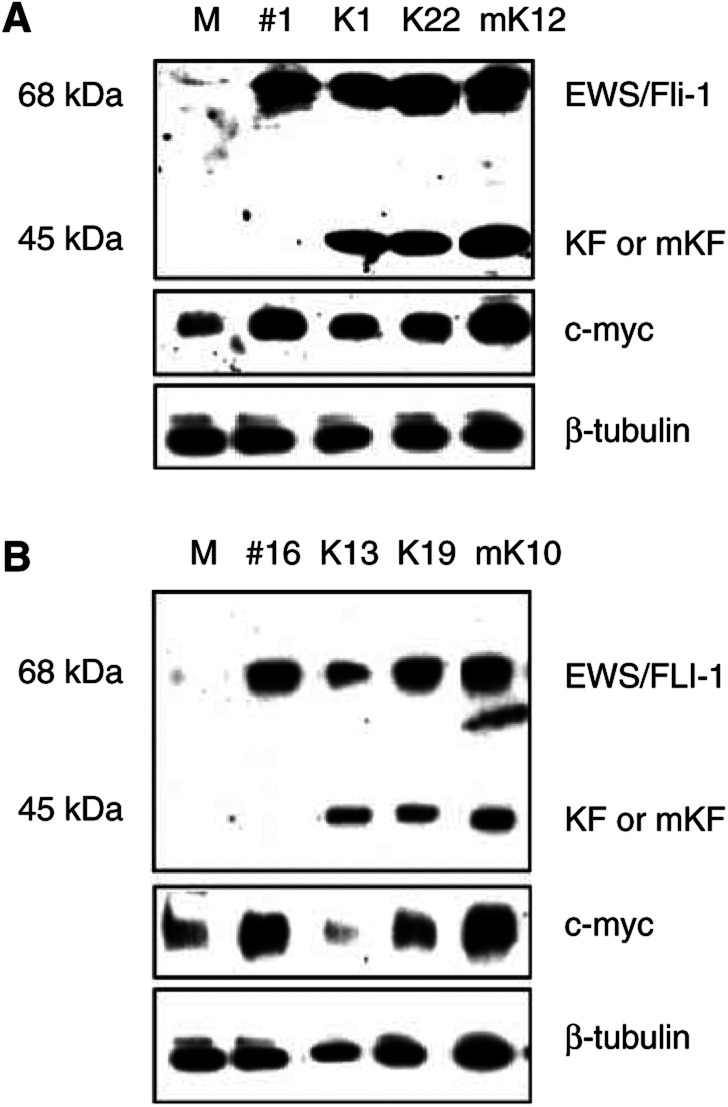
Western blot showing the expression of EWS/FLI-1 (68 kDa), KRAB/FLI-1 or mutant KRAB/FLI-1 (45 kDa), and alteration of c-myc protein levels in transfected NIH3T3 cells. (**A**) Cells transfected with empty construct (M=mock control) or murine EWS/Fli-1 (#1) and subclones of #1 cotransfected with KRAB/FLI-1 (K1, K22) or mutant KRAB/FLI-1 (mK12). (**B**) Similar human EWS/FLI-1-transformed clones transfected with KRAB/FLI-1 (K13, K19) or mutant KRAB/FLI-1 (mK10).

Murine and human EWS/FLI-1-transformed clones were used to test the effects of the KRAB/FLI-1 hybrid protein on the transformed phenotype. *KRAB/FLI-1* and mutant *KRAB/FLI-1* vectors were generated such that they contained the equivalent region of the FLI-1 DBD as that found in human type I *EWS/FLI-1* translocations and our human and mouse EWS/FLI-1 constructs. The mutant KRAB domain, which was used as a control, contains two amino-acid substitutions which abolishes KRAB binding to the corepressor KAP-1 and thus repressor function (Margolin *et al*, 1994; Friedman *et al*, 1996). Both *KRAB/FLI-1* and *mutant KRAB/FLI-1* fusion genes were placed under the control of the human EF-1α promoter (Figure 2). These genes were followed by a promoterless IRES/neomycin cassette to ensure that all G418-resistant clones expressed the *KRAB/FLI-1* or *mutant KRAB/FLI-1* fusion genes. Protein expression in G418-resistant clones was demonstrated by Western blot with the Fli-1 polyclonal antibody that detected both the 68 kDa EWS/FLI-1 protein band and the 45 kDa KRAB/FLI-1 or mutant KRAB/FLI-1 protein bands. Three murine and human EWS/FLI-1-transformed clones were each transfected with *KRAB/FLI-1* and data from two representative clones, mEF#1 and HuEF#16, are shown in Figure 3A

and 3B. Clones K1, K22 and mK12 are KRAB/FLI-1 and mutant KRAB/FLI-1 cotransfectants, respectively, of the mouse EWS/Fli-1-transformed clone mEF#1 (Figure 3A). These clones had similar EWS/Fli-1 protein levels to the parental mEF#1 relative to β-tubulin controls. Clone mK12 displayed a slightly higher expression of mutant KRAB/FLI-1 protein levels compared with K1 and K22 cotransfectants. Similar expression of human EWS/FLI-1, KRAB/FLI-1 and mutant KRAB/FLI-1 was observed in cotransfectants of the human EWS/FLI-1-transformed clone HuEF#16 (Figure 3B). These data indicate KRAB/FLI-1 and mutant KRAB/FLI-1 proteins are expressed at similar levels in these clones, and thus differences in the transformed phenotypes of these clones is likely to be a protein function.

### KRAB/FLI-1 reduces the proliferation rate of human or murine EWS/FLI-1 transformed NIH3T3 cells in low serum media

**Figure 4:**
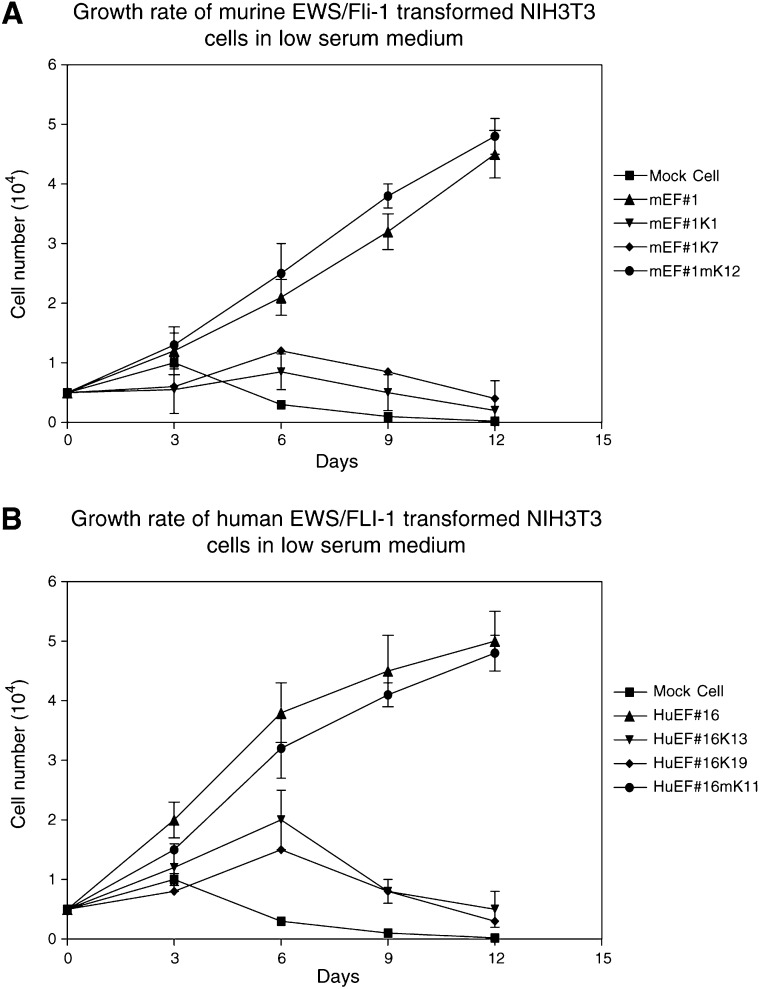
KRAB/FLI-1 inhibits the growth rate of EWS/FLI-1-transformed cells in low serum medium. In all, 5000 cells were grown in 1% FCS and cells were counted in triplicate at 3-day intervals and mean±s.d. is shown. (**A**) The parental murine Ews/Fli-1-transformed NIH3T3 cells (mEF#1) and KRAB/FLI-1 (K1, K7) or mutant KRAB/FLI-1 (mKF12) cotransfected subclones. (**B**) The parental human EWS/FLI-1-transformed NIH3T3 cells (HuEF#16) and KRAB/FLI-1 (K13, K19) or mutant KRAB/FLI-1 (mK11) cotransfected subclones. NIH3T3 cells transfected with empty vector were used as controls. Data are representative of three separate experiments (mean±s.d.).

Under normal culture conditions with 10% FCS there was no difference in the growth rate of clones expressing *EWS/Fli-1* and/or *KRAB/FLI-1* or *mutant KRAB/FLI-1* fusion genes (data not shown). KRAB/FLI-1 also had no effect on the growth of wild-type NIH3T3 cells. However, under low serum culture conditions (1% FCS), the EWS/FLI-1-transformed cell lines and clones coexpressing *mutant KRAB/FLI-1* grew rapidly (Figure 4A

and 4B), whereas EWS/FLI-1-transformed cells coexpressing *KRAB/FLI-1* had significantly slower or no growth (Figure 4A and 4B). The growth rate of these *KRAB/FLI-1* cotransfectants was similar to the control cell lines that did not express either fusion construct (Figure 4A and 4B). Thus, *KRAB/FLI-1* was able to inhibit the growth of both human or murine EWS/FLI-1-transformed cells.

### KRAB/FLI-1 reduces the colony formation of murine and human EWS/FLI-1-transformed NIH3T3 cells in soft agar

**Figure 5:**
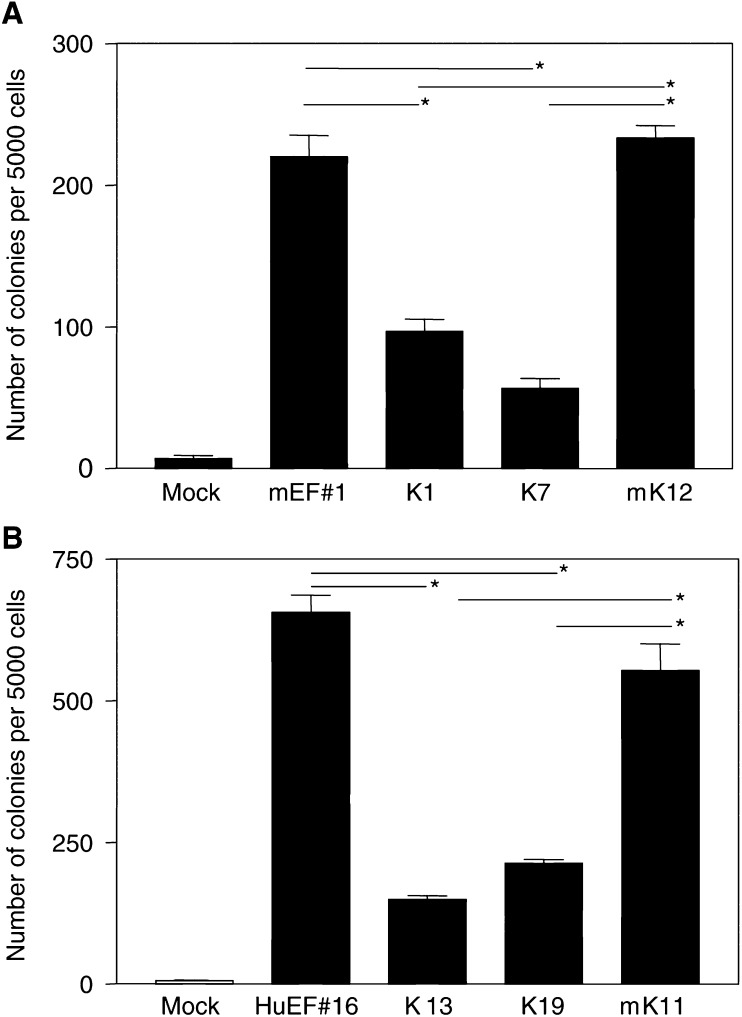
KRAB/FLI-1 inhibits colony formation in soft-agar assays. (**A**) The parental murine Ews/Fli-1-transformed NIH3T3 cells (mEF#1) and KRAB/FLI-1 (K1, K7) or mutant KRAB/FLI-1 (mK12) cotransfected subclones. (**B**) The parental human EWS/FLI-1-transformed NIH3T3 cells (HuEF#16) and KRAB/FLI-1 (K13, K19) or mutant KRAB/FLI-1 (mK11) cotransfected subclones. Data are representative of three separate experiments and values shown are the mean±s.e.m. of triplicate samples at day 12. Samples indicated (^*^) were significantly different (*P*<0.05).

The ability of KRAB/FLI-1 to reverse the transformation phenotype of EWS/FLI-1-expressing cells was also tested using the soft agar assay. Human or murine EWS/FLI-1-transformed NIH3T3 cell clones were tested in triplicate in three separated experiments. Data from two representative *EWS/FLI-1* expressing clones (one human, HuEF#16, and one murine, mEF#1) are shown in Figure 5A

and 5B. After 2 weeks, the parental human and mouse EWS/FLI-1-transformed NIH3T3 cells formed ∼650 and ∼200 colonies of >20 cells, respectively (Figure 5A and 5B). Clones cotransfected with mutant KRAB/FLI-1 (mEF#1mK12 and HuEF#16mK11), showed no significant inhibition of soft-agar growth, however, clones cotransfected with KRAB/FLI-1 (mEF#1K1 and mEF#1K7, and HuEF#16K13 and HuEF#16K19) showed a significant reduction in the number of colonies formed (Figure 5A and 5B). These data indicate that the KRAB/FLI-1 repressor impairs anchorage-dependent growth of EWS/FLI-1-transformed cells.

### KRAB/FLI-1 impairs tumour development of murine and human EWS/FLI-1-transformed NIH3T3 cells in nude mice

**Figure 6:**
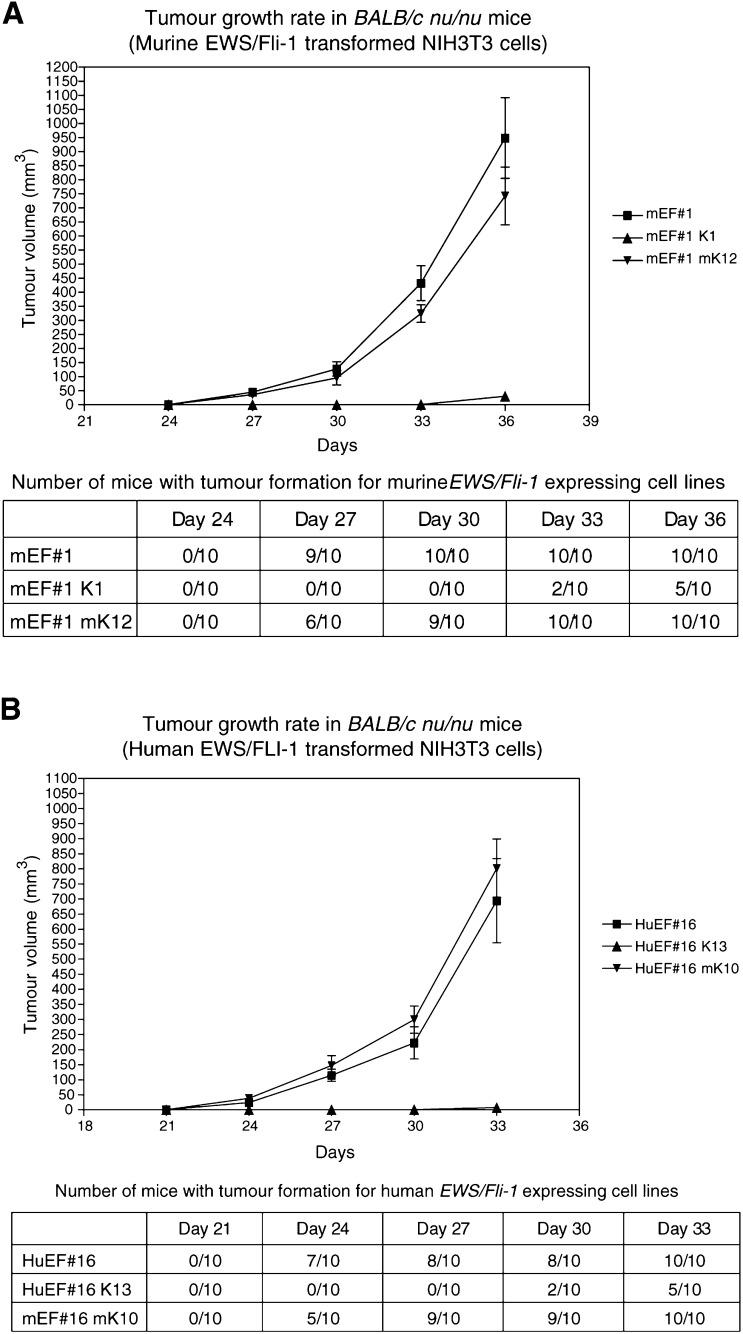
KRAB/FLI-1 inhibits the growth of tumours in nude mice. Cells (1×10^6^) were inoculated into the flanks of *BALB/c nu/nu* mice and tumours measured with calipers at 3-day intervals. (**A**) The parental murine Ews/Fli-1-transformed NIH3T3 cells (mEF#1) and KRAB/FLI-1 (K1) or mutant KRAB/FLI-1 (mK12) cotransfected subclones. (**B**) The parental human EWS/FLI-1-transformed NIH3T3 cells (HuEF#16) and KRAB/FLI-1 (K13) or mutant KRAB/FLI-1 (mK10) cotransfected subclones. Mean tumour volumes (±s.e.m.) are shown and were calculated by the formula (mean diameter)^3^×π/6. The table shows the number of inoculation sites forming tumours at each time point.

For each murine or human EWS/FLI-1-transformed clone, one KRAB/FLI-1 cotransfectant and one mutant KRAB/Fli-1 cotransfectant were inoculated into *BALB/c* *nu/nu* mice. Control cells were not observed to form any tumours (data not shown). The HuEF#16-transformed clone formed tumours of 222±53 mm^3^ by 30 days after inoculation, whereas the mEF#1 transformed clone had formed tumours of only 128±26 mm^3^ at the same stage (Figure 6A

and 6B). The faster tumour growth rate of this clone HuEF#16 was consistent with its more rapid growth rate observed *in vitro* and higher expression of EWS/FLI-1. Clones that expressed both mutant KRAB/FLI-1 and EWS/FLI-1 displayed a similar tumour growth rate in nude mice to that of the parental EWS/FLI-1-expressing cells; however, clones that expressed KRAB/FLI-1 showed significantly reduced tumour size (Figure 6A and 6B). For example, mouse Ews/Fli-1-transformed cells coexpressing mutant KRAB/FLI-1 formed tumours in nine out of 10 mice inoculated with a mean size of 96±26 mm^3^ by day 30; however, at the same stage no tumours were observed in cells coexpressing KRAB/FLI-1. Similarly, in mice inoculated with human EWS/FLI-1 clone #16 coexpressing KRAB/FLI-1, no tumours were observed at day 27; however, tumours formed in nine out of 10 mice inoculated with the same clone expressing mutant KRAB/FLI-1 with a mean volume of 147±32 mm^3^. In all 50% of the mice inoculated with clones expressing both KRAB/FLI-1 and EWS/FLI-1 did develop small palpable tumours (7±2 mm^3^) in the latter part of the experiment (Figure 6A and 6B); however, these tumours continued to grow very slowly (data not shown).

### KRAB/FLI-1 suppresses the transformed phenotype of SK-N-MC, a PNET cell line

**Figure 7:**
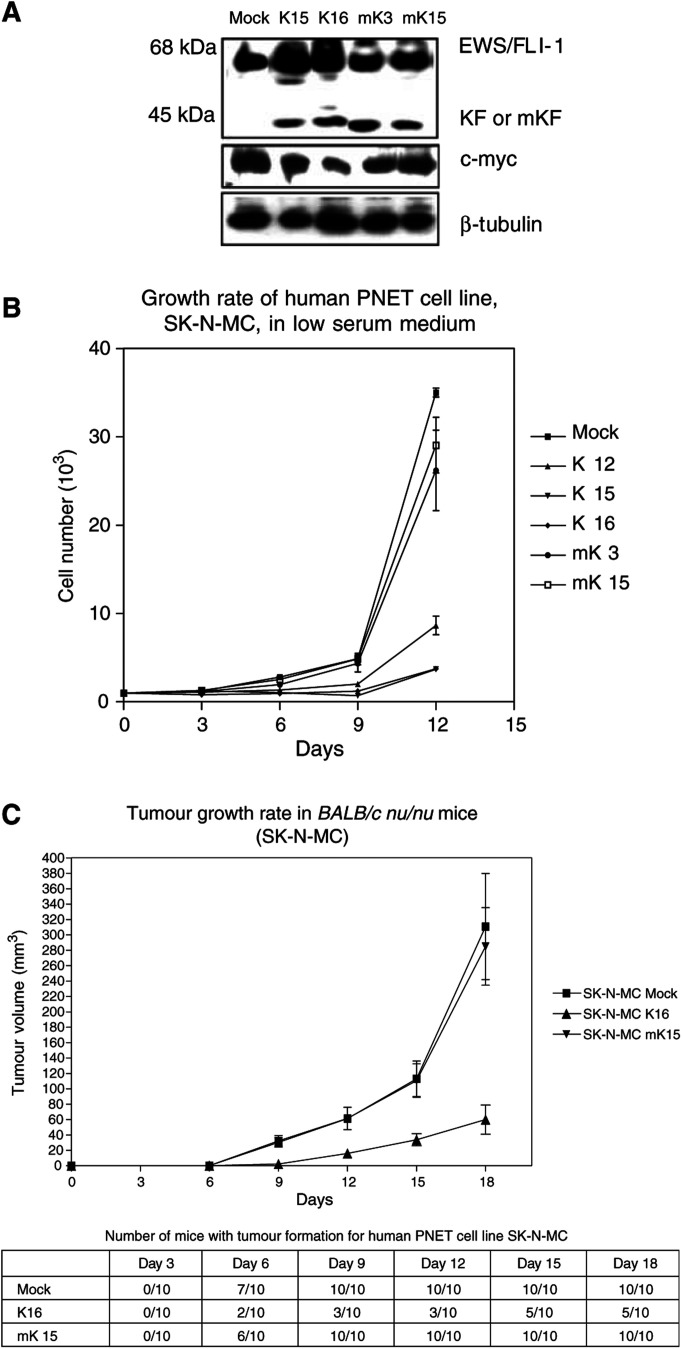
KRAB/FLI-1 inhibits transformed phenotype of a human PNET cell line (**A**) Western blot showing the expression of EWS/FLI-1, KRAB/FLI-1 or mutant KRAB/FLI-1 and c-myc protein levels. (**B**) Growth rate in low serum media. Cells (1×10^3^) were seeded in triplicate in media supplemented with 1% FCS and counted at 3-day intervals. Data are shown as mean±s.d. K12, K15 and K16 were KRAB/FLI-1-expressing clones while mK3 and mK15 were mutant KRAB/FLI-1 expressing clones. Mock indicates the empty vector transfected control. (**C**) The tumour growth rate in *BALB/c nu/nu* mice. Cells (1×10^5^) were inoculated into the flanks of *BALB/c nu/nu* mice and palpable tumours were measured with calipers at 3-day intervals. Mean tumour volumes (+s.e.m.) are shown and were calculated by the formula (mean diameter)^3^×*π*/6. The table shows the number of inoculation sites forming tumours at each time point.

Our data demonstrate that human and mouse EWS/FLI-1 transforms NIH3T3 cells and this transformation could be repressed by KRAB/FLI-1. Since the precise aetiology of ES/PNET is not defined and, like other cancers, these tumours contain other mutations, we were interested to determine whether KRAB/FLI-1 could also suppress the transformed phenotype of a human PNET cell line. Thus, we transfected *KRAB/FLI-1* (or *mutant KRAB/FLI-1* as a control) into the human PNET cell line SK-N-MC. This cell line has a type I EWS/FLI-1 translocation similar to our constructs generated above, but also has a defined mutation in the tumour suppressor p53 (Beerli *et al*, 1998). Clones were generated and expression of both EWS/FLI-1 (68 kDa) and KRAB/FLI-1 or mutant KRAB/FLI-1 (45 kDa) proteins was examined by Western blot (Figure 7A

). Unlike our NIH3T3 transfectants, the amount of EWS/Fli-1 fusion protein was higher than that of the KRAB/FLI-1/mutant KRAB/FLI-1 chimaeric proteins.

To assess the ability of KRAB/FLI-1 to inhibit transformation of SK-N-MC cells *in* *vitro*, we determined their growth rates in media with low serum. KRAB/FLI-1-expressing clones proliferated at a slower rate compared to both mutant KRAB/FLI-1-expressing clones and control SK-N-MC cells (Figure 7B). Unlike NIH3T3 cells coexpressing KRAB/FLI-1 and EWS/FLI, KRAB/FLI-1-expressing SK-N-MC cells continued to proliferate suggesting that the KRAB/FLI-1 repressor could not completely abolish the transformed phenotype of SK-N-MC cells, perhaps because of the relative levels of KRAB/FLI-1 and EWS/FLI-1 proteins.

To determine the ability of the KRAB/FLI-1 repressor to influence tumour development of SK-N-MC cells, we inoculated clones of SK-N-MC cells expressing KRAB/FLI-1 or mutant KRAB/FLI-1 and control cells into nude mice as described above. The mean tumour volume was significantly reduced in KRAB/FLI-1 expressing SK-N-MC cells compared with both mock-transfected and mutant KRAB/FLI-1-expressing cells (Figure 7C). For example, at day 15 100% of mice had developed tumours in the parental and mutant KRAB/FLI-1-expressing cells (mean tumour volume 113±23 and 111±22 mm^3^, respectively), whereas only 50% of mice inoculated with KRAB/FLI-1-expressing cells had palpable tumours (mean volume 34±8 mm^3^). These data indicate that the KRAB/FLI-1 repressor could significantly reduce tumour development, similar to the *in vitro* data.

## DISCUSSION

The majority of Ewing's sarcoma and primitive neuroectodermal tumours are associated with a t(11;22)(q24;q12) chromosomal translocation, which results in the production of an EWS/FLI-1 fusion protein that comprises the amino terminus of the *EWS* gene and the carboxyl terminus of the *FLI-1* gene. The continued expression of human EWS/FLI-1 protein has been shown to be sufficient and necessary for *in vitro* transformation of cell lines (May *et al*, 1993a,1993b). Although EWS/FLI-1 has been shown to be a potent transcription factor (Delattre *et al*, 1992; May *et al*, 1993a,1993b; Bailly *et al*, 1994 and references therein), whether its ability to transform is due only to its transcriptional activation activity has been unclear. Furthermore, its importance in the aetiology of human disease is also unclear as these human tumours are likely to contain multiple mutations. To address these issues, we have demonstrated herein that both human and murine EWS/FLI-1 can transform NIH3T3 cells and that the transformation phenotype in these cells and a human ES/PNET cell line can be inhibited by a specific transcriptional repressor KRAB/FLI-1. The latter also sheds light on the mechanism of EWS/FLI-1 transformation since the FLI-1 DBD is necessary to target an active repressor domain to the effector site.

In order to facilitate mouse model studies, we engineered a murine *Ews/Fli-1* fusion gene to mimic the human fusion gene formed by the most common chromosomal translocation, t(11;22)(q24;q12), observed in ES/PNET. This murine fusion gene showed 98% nucleotide identity to the human fusion gene and fibroblast transfectants showed similar transformed phenotypes to human EWS/FLI-1-transfected cells. This included anchorage-independent growth in soft agar, increased proliferation rate in low serum media and tumour development in *BALB/c nu/nu* mice. Variations in the efficiency of clones to display a transformed phenotype appeared to correlate with the level of the EWS/FLI-1 fusion proteins rather than the species from which the sequences were derived. These findings validate at a protein level previous reports that suggested a correlation between the mRNA levels of human EWS/FLI-1 and the proliferation rate of ES/PNET cells (Tanaka *et al*, 1997). These data indicate the feasibility and validity of a murine model for ES/PNET, which would provide a valuable resource for elucidating the mechanisms of EWS/FLI-1 transformation and the development of a model in which to test novel therapeutic strategies.

To inhibit EWS/FLI-1 transactivation activity in both human and mouse EWS/FLI-1-transformed cells and a human PNET cell line, we generated a KRAB/FLI-1 chimaeric protein. When bound to DNA by a specific DBD, the KRAB domain binds to the corepressor KAP-1, which suppresses transcription by either directly inhibiting the transcriptional machinery and/or by altering the chromatin structure (Friedman *et al*, 1996; Le Douarin *et al*, 1996; Moosmann *et al*, 1996; Darnell and Richardson, 1999). Indeed, KRAB/FLI-1 inhibited EWS/FLI-1 transformation in our study. Since KRAB/FLI-1 will only inhibit transcription of genes with FLI-1 DNA-binding elements and will not have any effect on the nontransactivation activity of EWS/FLI-1, these data demonstrate that transactivation of FLI-1 target genes by EWS/FLI-1 is essential for its ability to transform. This is in contrast with previous data, which indicated that mutation of the EWS/FLI-1 DBD did not eliminate its ability to transform (Jaishankar *et al*, 1999; Welford *et al*, 2001). We suggest that the remaining transformation activity reported for the mutant EWS/FLI-1 could be explained by the ability of the fusion protein to still complex with the transcription initiation complex independent of DNA-binding and/or residual-DNA-binding activity not detected in *in vitro* assays.

Our observation that suppression of EWS/FLI-1-activated genes inhibits transformation *in vitro* and *in vivo* is consistent with previous studies, which demonstrated that expression of a FLI-1 DBD can suppress Ewing's sarcoma and EWS/FLI-1-transformed cell growth *in vitro* (Kovar *et al*, 1996; Welford *et al*, 2001), presumably by competing with EWS/FLI-1 DNA binding. However, in Welford *et al*,(2001) expression of this FLI-1 DBD did not affect tumour growth of EWS/FLI-1-transformed NIH3T3 cells *in vivo.* This was suggested to be a result of DBD-independent effects of the EWS/FLI-1 protein, although inhibition of an ES cell line was observed (Welford *et al*, 2001). It is possible that the levels of the human FLI-1 DBD protein used were not sufficient to inhibit EWS/FLI-1 transactivation in murine NIH3T3 cells *in vivo*. This is supported by our study where the active repressor KRAB/FLI-1 efficiently inhibited transformed cell growth *in vitro* and *in vivo*, whereas similar levels of mutant KRAB/FLI-1 had no significant effect on the transformed phenotype. It is logical to conclude that levels of an active repressor required to inhibit transcription would be much lower than those of a DNA-binding competitor.

The inhibition of EWS/FLI-1 is the first demonstration of the KRAB domain suppressing the action of an ETS factor. Many members of this large family of transcription factors have also been implicated as potential oncogenes in other malignancies including acute lymphoblastic leukaemia, myelomonocytic leukaemias and breast cancer (Santoro *et al*, 1992; Ichikawa *et al*, 1994; Golub *et al*, 1995; Chen *et al*, 1996; Tymms *et al*, 1997). Thus, similar use of DNA sequence-specific transcriptional repressors will be an important avenue for the development of broader therapeutic strategies.

The use of a targeted and potent repressor domain increases the efficiency of transcriptional repression of cancer-causing genes. Thus, an active transcriptional repressor is more efficient for the inhibition of tumour growth than overexpression of a DBD. Effective therapies will thus be possible with much lower amounts of protein.

A major issue in understanding the aetiology of ES/PNET is understanding how EWS/FLI-1 activates the many genes shown to be increased. Since the promoters of some of the genes activated in ES/PNET are believed to not bind FLI-1, including c-myc, it has been suggested that the EWS/FLI-1 protein may have additional properties such as RNA processing (Jaishankar *et al*, 1999) or the binding and modulation of genes not normally regulated by FLI-1 (May *et al*, 1993a). Significantly, c-myc was increased in each of our cell lines expressing human or mouse EWS/FLI-1 and decreased in clones coexpressing KRAB/FLI-1. Since the KRAB domain shows no DNA-binding activity (Margolin *et al*, 1994) the regulation of this gene together with transformed phenotype appears to be a result of FLI-1-specific DNA binding. This suggests that the many genes that are activated by EWS/FLI-1, but not by direct FLI-1 DNA binding, may be indirectly activated. The mechanism by which indirect activation occurs is unclear; however, the analysis of KRAB/FLI-1 expressing ES/PNET cell lines will enable the identification of genes specifically regulated through FLI-1-specific DNA binding in ES/PNET. The identification of the primary target genes of EWS/FLI-1 will be important in understanding the oncogenic processes in ES/PNET and will further assist in the development of new therapeutic strategies.
